# Do *GST* Polymorphisms Modulate the Frequency of Chromosomal Aberrations in Healthy Subjects?

**DOI:** 10.1289/ehp.0900838

**Published:** 2009-09

**Authors:** Pavel Vodicka, Alessio Naccarati, Ludmila Vodickova, Veronika Polakova, Maria Dusinska, Ludovit Musak, Erika Halasova, Simona Susova, Pavel Soucek, Kari Hemminki

**Affiliations:** Institute of Experimental Medicine, Academy of Sciences of the Czech Republic, Prague, Czech Republic, E-mail: pvodicka@biomed.cas.cz; Slovak Medical University, Bratislava, Slovak Republic; Jessenius Faculty of Medicine, Martin, Slovak Republic; National Institute of Public Health, Prague, Czech Republic; German Cancer Research Center, Heidelberg, Germany

Rossi et al. (2009) described an association between chromosomal aberration (CA) frequency and cancer risk in a case–control study on 107 cancer cases and 291 controls, whereby they observed no modifying effect of polymorphisms in glutathione *S-*transferase M1 (*GSTM1*) and *GSTT1.*

In our studies of 488 healthy individuals who shared the same environmental exposure in Slovakia and the Czech Republic, we observed a CA frequency of 2.35 ± 1.73 (mean ± SD) (Halasova et al. 2007; Musak et al. 2008; Naccarati et al. 2006; Slyskova et al. 2007; Vodicka et al. 2001, 2004a, 2004b). The frequencies (mean ± SD) for chromatid-type abberations (CTA) and chromosome-type aberrations (CSA) were 1.22 ± 1.21 and 1.15 ± 1.35, respectively. By analyzing modulating effects of genetic polymorphisms in *GSTT1, GSTM1,* and *GSTP1* on CAs, CTAs, and CSAs (Table 1), we found no significant association between chromosomal damage and any of the studied polymorphisms. The results were further confirmed by logistic regression: for the *GSTT1* null genotype, odds ratio (OR) = 1.35 [95% confidence interval (CI), 0.79–2.32; *p* = 0.27]; for the *GSTM1* genotype, OR = 1.09 [95% CI, 0.74–1.62; *p* = 0.65]; and for a variant *GSTP1* Val105Val genotype, OR = 0.83 [95% CI, 0.55–1.24; *p* = 0.36]. These data on a larger healthy population [previously published separately by Halasova et al. (2007), Musak et al. (2008), Naccarati et al. (2006), Slyskova et al. (2007), and Vodicka et al. (2001, 2004a, 2004b)] confirm the findings of Rossi et al. (2009) regarding *GSTM1* and *GSTT1* polymorphisms. Additionally, in our reanalysis, we did not observe any modulating effect of *GSTP1* polymorphism on CA frequency. However, the modulating role of *GST* polymorphisms may not be excluded, particularly in interaction with heavy occupational exposure. In our study exploring chromosomal damage in tire-plant workers (Musak et al. 2008), CAs were significantly higher in subjects with *GSTT1*-null than in those with *GSTT1*-plus genotypes, particularly in association with smoking.

In the past decade, CAs have been accepted as a predictive marker of cancer (Hagmar et al. 2004), particularly for colo-rectal and lung cancers (Boffetta et al. 2007; Norppa et al. 2006). Nevertheless, these studies, as well as the study of Rossi et al. (2009) may have limitations: For example, cohorts were recruited in various regions with different lifestyle and environmental backgrounds, and different laboratories were involved in processing and scoring the samples over many years. In earlier studies, virtually no data on individual susceptibility were available because of the lack of DNA for molecular analysis.

The data on CAs presented here were obtained on healthy subjects from a homogeneous region with fairly similar socioeconomic background. The analysis of CAs reported in these studies (Halasova et al. 2007; Musak et al. 2008; Naccarati et al. 2006; Slyskova et al. 2007; Vodicka et al. 2001, 2004a, 2004b**) were performed in two labo**ratories, using the same protocol and the same scoring criteria with regular slide exchanges to minimize interlaboratory and inter scorer differences. Also, native DNA from whole-blood samples for molecular genetic studies was collected simultaneously with the samples for cytogenetic investigations.

Future prospective studies regarding CAs and cancer should be designed by taking into account the lifestyle and occupational/environmental exposures, along with factors of individual susceptibility. Some *GST* polymorphisms may modulate CA frequency through interaction with environmental factors. The next logical step for a confirmation of predictive values of CA frequencies in relation to cancer will be their determination in lymphocytes of cancer patients in association with clinical– pathological characteristics.

## Figures and Tables

**Table 1:** Distribution of analyzed genotypes and CA frequencies.

		CA			CTA			CSA		
Genotype	No.	Mean ± SD	χ^2^	*p*-Value	Mean ± SD	χ^2^	*p*-Value	Mean ± SD	χ^2^	*p*-Value
*GSTT1* deletion										
Plus	356	2.26 ± 1.71	1.59	0.21	1.20 ± 1.21	0.88	0.35	1.11 ± 1.28	1.01	0.31
Null	74	2.59 ± 1.84			1.34 ± 1.29			1.29 ± 1.47		

*GSTM1* deletion										
Plus	223	2.28 ± 1.77	0.43	0.51	1.25 ± 1.28	0.01	0.90	1.04 ± 1.32	1.11	0.29
Null	208	2.36 ± 1.70			1.20 ± 1.17			1.18 ± 1.38		

*GSTP1* Ile105Val										
Ile/Ile	176	2.42 ± 1.91	2.25	0.33	1.31 ± 1.38	0.36	0.84	1.12 ± 1.34	1.27	0.53
Ile/Val	219	2.19 ± 1.60			1.14 ± 1.08			1.07 ± 1.36		
Val/Val	36	2.58 ± 1.61			1.31 ± 1.31			1.28 ± 1.37		

Data, pooled and recalculated from previously published data (Halasova et al. 2007; Musak et al. 2008; Naccarati et al. 2006; Slyskova et al. 2007; Vodicka et al. 2001, 2004a, 2004b), were analyzed by the Kruskal-Wallis test.
